# Embedding behavioural and implementation science in a whole school approach to promote physical activity among children and young people with disabilities

**DOI:** 10.1371/journal.pone.0362562

**Published:** 2026-11-13

**Authors:** Erin Byrd, Jason J. Wilson, Paul McFlynn, Victoria Simms, Laurence Taggart, Áine Boner, Marie H. Murphy

**Affiliations:** 1 School of Sport and Exercise Science, Faculty of Life and Health Sciences, Ulster University, Belfast, Northern Ireland, United Kingdom; 2 School of Education, Faculty of Arts, Humanities and Social Sciences, Ulster University, Belfast, Northern Ireland, United Kingdom; 3 Centre for Mathematical Cognition, School of Science, Loughborough University, England, United Kingdom; 4 School of Nursing and Midwifery, Faculty of Medicine, Health and Life Sciences, Queens University Belfast, Belfast, Northern Ireland, United Kingdom; 5 Physical Activity for Health Centre, Moray House School of Education and Sport, University of Edinburgh, Scotland, United Kingdom; Nagoya University: Nagoya Daigaku, JAPAN

## Abstract

**Background:**

A whole school approach (WSA) to physical activity is a comprehensive strategy encouraging children's physical activity throughout the school day. However, research on implementing this approach in special educational needs (SEN) school settings is limited. A strategic approach that considers the school context, behaviour change mechanisms and implementation strategies can improve the effectiveness and sustainability of physical activity in a WSA. This study aimed to embed implementation and behavioural science principles to co-develop an implementation research logic model (IRLM) to guide implementation of a WSA to physical activity in SEN settings.

**Methods:**

A collaborative co-development approach was used in this study exploring the WSA to physical activity in SEN settings. Teachers, school leadership, parents, the education authority and professionals from community sports clubs in one UK region participated in a series of workshops. The workshops and analyses were guided by behavioural and implementation theories, models, and frameworks.

**Results:**

Workshop discussions revealed 31 determinants of implementation, comprising 18 facilitators, 12 barriers and one item identified as both barrier and facilitator. These determinants informed the development of eight implementation strategies to promote physical activity through a WSA in SEN settings (e.g., staff training, building collaborative networks and engaging families). Eleven implementation, seven school-level and six pupil-level outcomes, were identified to assess effectiveness and implementation. Eight causal mechanisms explained the pathway from strategies to outcomes. Finally, these determinants, implementation strategies, mechanisms, and outcomes were mapped onto an IRLM to guide the implementation of a WSA to physical activity for children and young people with SENs across settings.

**Conclusions:**

We have co-developed an IRLM embedded in implementation and behavioural science to support the implementation of a WSA to physical activity in SEN settings. The model illustrates causal mechanisms between implementation determinants and implementation strategies to achieve intended outcomes. This IRLM has informed a guidance document for implementing WSA to physical activity within SEN settings, addressing real-world challenges faced in promoting physical activity among pupils with SEN.

## Introduction

Children and young people with additional needs and disabilities engage in lower levels of physical activity than their typically developing peers, and as a result, experience a greater risk for numerous physical and mental health comorbidities [1]. Physical activity behaviours are established early in life [2] and have been shown to track with moderate-high stability through adolescence into adulthood [3]. As children spend a large proportion of their waking time in school, this setting is crucial for promoting physical activity and solidifying lifelong movement behaviours [4]. Schools have been identified as a preferred setting for physical activity interventions for children and young people, with increasing evidence on the effectiveness of multi-component interventions [5].

### Whole school approaches to physical activity

National and international guidance now recommends the adoption of Whole School Approaches (WSA) to physical activity, a strategy that encompasses all aspects of a school environment, including physical and cultural factors, to promote and support physical activity among students [6,7].

These comprehensive WSAs help us to understand the complexities involved in promoting physical activity within schools, maximises partnership action, and leverages policy for change [8]. A WSA encompasses three interrelated pillars: curriculum, teaching and learning; the school ethos and environment; and family and community partnerships [9]. Therefore, interventions utilising a WSA are woven into daily classroom routines and curriculum and extend to out-of-class activities. They involve all school staff, foster parent engagement and collaboration, and coordinate with external services and agencies [10]. However, studies exploring the implementation of WSA in Special Education Needs (SEN) school settings are lacking.

### Considering context in the whole school approach to physical activity

The specific contexts of individual schools, including physical environment, staff, and sociodemographic factors, have been shown to considerably impact the effectiveness and implementation of WSA to physical activity interventions within mainstream educational settings [11]. By engaging key school community stakeholders within a WSA, a more comprehensive and sustainable strategy can be developed that ensures an intervention is tailored to the specific needs and context of the school community [8,12]. Additionally, this collaboration fosters a sense of ownership and investment in the intervention and increases the likelihood of successful implementation and long-term impact on pupils’ physical activity levels [11]. Identifying barriers or challenges that may arise during the implementation phase, allows for proactive problem-solving and adjustment of strategies to maximise potential for success [12].

In the SEN context, the unique and greater diversity of pupil need, different challenges faced by school staff, and the flexible application of the school curriculum [13], emphasises the requirement to identify the specific contextual needs and determinants facing this setting when implementing a WSA. In Northern Ireland special education schools provide specialist education for pupils with a range of special educational needs, including learning or intellectual disability, autistic spectrum disorder, physical and sensory impairments, speech, language and communication needs, and complex or multiple needs requiring additional educational support. This diversity of educational and support needs has important implications for implementation, as physical activity interventions must often be adapted to accommodate differences in pupils’ cognitive, communication, sensory, physical, and behavioural profiles while remaining feasible and sustainable within the school environment.

### Frameworks and toolkits for promoting a whole school approach to physical activity

Various frameworks and toolkits have been developed to support schools to implement WSA to physical activity. These toolkits guide schools in implementing comprehensive physical activity programmes and strategies, emphasising the targeting of multiple levels of influence for effectiveness, and aid prioritisation and planning of school-wide physical activity, allowing schools to decide priorities and feasibility of approaches within their context [14–17]. By following these guides, schools can create a holistic environment that promotes physical activity through the entire school system, connecting with the home environment and wider community. These toolkits, however, were developed to target mainstream schools, and have not yet been adapted, applied or evaluated within SEN school settings. Therefore, their applicability, relevance, and utility within a SEN setting, considering the unique contextual needs of the special education schools and pupils, cannot be determined.

### Use of implementation science in promoting a whole school approach to physical activity

When the contextual determinants, processes and rationale behind implementing a new practice or physical activity initiative are not clearly understood, it becomes difficult to identify which factors contributed to successful adoption and implementation [18]. Therefore, using systematic approaches and frameworks during implementation planning is important to ensure success and sustainability [19]. This systematic approach will allow for a better understanding of how implementation strategies work, for whom they are effective, under what specific conditions, and how they influence both implementation success and health outcomes [20].

A major challenge when applying this approach to implementation planning is the existence of different models and frameworks that attempt to describe and understand the complex process of changing practices and integrating evidence-based interventions across organisations such as schools’ settings [21]. These frameworks usually focus on aspects such as understanding the 1) contextual background; 2) identifying the barriers and facilitators to implementing the intervention (e.g., Consolidated Framework for Implementation Research (CIFR 2.0) [22]), 3) describing the strategies used and steps involved in the process (e.g., Expert Recommendations for Implementing Change (ERIC) [23]); and 4) evaluating outcomes (e.g., Implementation Outcomes Framework (IOF) [24]). While each framework holds its own value in identifying and articulating core constructs of the implementation process, trying to combine multiple frameworks (e.g., CFIR, ERIC, IOF) can sometimes lead to construct collision or overlap. The challenge arises from blending these frameworks into a comprehensive map, pathway, or logic model to support ‘real-world’ implementation without losing the clarity required for usability.

### Theoretical underpinning

With this complexity in mind, several theoretical frameworks underpinned the current study to inform the different elements of the map, pathway or Implementation Research Logic Model (IRLM).

*The CFIR Framework:* The updated Consolidated Framework for Implementation Research (CFIR 2.0) [25] was used to explore barriers and facilitators to implementing a WSA to physical activity. The CFIR is a conceptual model that helps understand the factors influencing the implementation of complex interventions. It encompasses five domains: Innovation Characteristics, Outer Setting, Inner Setting, Characteristics of Individuals, and Implementation Process. The CFIR framework allows for the exploration of critical factors that might affect acceptability, adoption, and sustainability of a WSA to physical activity in SEN settings. CFIR encourages a consideration of various contextual factors, such as school and community readiness, environmental influences, and individual beliefs, which can impact the success of WSA to physical activity initiatives. The CFIR framework enables the identification of barriers and facilitators from multiple perspectives, allowing a deeper understanding of the complex SEN setting environment.

*ERIC:* The Expert Recommendations for Implementing Change (ERIC) [23] was used to categorise implementation strategies within the IRLM. The ERIC framework provides a list of 73 discrete implementation strategies spanning nine strategy domains [23]. The ERIC framework was developed to help practitioners and researchers systematically report implementation strategies. The ERIC compilation of strategies can be viewed as building blocks to develop multifaceted strategies to address often complex determinants of implementation for a specific intervention [26].

*IOF:* For the implementation outcomes, Proctor and colleagues’ [24] Implementation Outcomes Framework (IOF) was used to explore items for monitoring and evaluating the effectiveness of the WSA to physical activity. The IOF categorises outcomes and indicators of success of implementation into three categories: 1) Implementation Outcomes, 2) Service-level outcomes (school), and 3) Individual Level Outcomes (pupil). The implementation outcomes taxonomy developed by Proctor and colleagues encompasses eight distinct but interrelated implementation outcomes including acceptability, adoption, appropriateness, cost, feasibility, fidelity, penetration and sustainability. Service outcomes include outcomes such as efficiency, safety, equity, and timeliness; and individual outcomes including satisfaction, function and symptomatology.

*Mechanisms of change theories:* Two additional theoretical frameworks were chosen to guide the complex task of developing potential mechanisms of change: the Normalisation Process Theory (NPT) [27] and the Capability, Opportunity, and Motivation model (COM-B) model of behaviour change [28]. These two frameworks were chosen to help explain both individual behaviour mediators and service-level causal mechanisms that would produce the intended implementation outcomes. The NPT supplies four implementation processes (Coherence, Cognitive Participation, Collective Action, and Reflexive Monitoring) that map directly to mechanisms of change, while COM-B specifies the proximal behavioural mechanisms (capability, opportunity, motivation) that drive individual behaviours that can influence adoption. For example, the creation of a shared folder with a range of movement break activities can create the physical opportunity for teachers to adopt activities in the classroom by providing easily accessible resources. Within the NPT, the four process constructs describe the social and cognitive work required to embed an intervention in the real-world settings. Positioning each as a mechanism of change clarifies the causal pathway from strategies to school-level outcomes [29]. The COM-B model identifies the immediate, internal, or situational factors that mediate whether implementation behaviours occur, and can be embedded as measurable mechanisms of individual behaviour change within an IRLM [30].

*Socio-ecological model:* Finally, throughout this study the Socio-Ecological Model (SEM) was applied to encourage a consideration of the whole school community, from the experiences of the pupil, school staff, families, the whole school as an organisation and the wider community. The SEM is a framework that recognises the multiple levels of influence on individual physical activity [31]. It highlights the interconnectedness of factors operating at various layers, including individual, interpersonal, organisational, community, and policy levels [32]. The application of the SEM facilitated comprehensive discussions by encouraging a consideration of influences beyond individual choices, exploring how broader social, environmental, and policy contexts shape physical activity behaviours and outcomes within the school.

### Use of implementation research logic models

Logic models are graphic depictions of the linear relationships among various elements of a programme or intervention and have been used for decades in complex intervention development [33]. More recently, the Implementation Research Logic Model (IRLM) was developed due to a recognition that in order to conduct implementation research, unique challenges needed to be addressed, and implementation-specific theories, models, and frameworks needed to be applied [34]. The IRLM specifies the relationships between the determinants of implementation, the implementation strategies, the mechanisms of action resulting from the strategies, and the outcomes affected: including implementation outcomes, service level outcomes, and individual level outcomes [35 (p157)].

### Aims of the study

The present study forms part of a larger project that aimed to co-develop, implement, and evaluate a whole school intervention to enhance movement and physical activity of pupils in special education settings. The wider project involved multiple stakeholder groups, including educators, school leaders, health professionals, parents, and pupils, with stakeholder involvement occurring at different stages of the research according to the aims of each phase. The current study reports the co-development of the intervention's Implementation Research Logic Model (IRLM), representing one component of the broader project. Therefore, this study aimed to develop an IRLM for implementing a WSA to physical activity for children and young people with additional needs and disabilities, grounded in behavioural and implementation science. The aim was achieved through the following objectives:

1. To utilise a collaborative co-development approach, and behavioural and implementation science frameworks, to identify determinants influencing physical activity in schools for children and young people with additional needs and disabilities.
2. To systematically map contextual and implementation determinants to strategies that will promote successful implementation of a WSA to physical activity in SEN settings.
3. To develop theoretical mechanisms of change, founded in behavioural science, of how implementation strategies address determinants and lead to intended outcomes of the WSA to physical activity.
4. To map the identified determinants, implementation strategies, mechanisms and outcomes to an Implementation Research Logic Model (IRLM).

For this study determinants are defined as the contextual, organisational, individual, and intervention-related factors that influence implementation. They can be categorised as either barriers or facilitators. Henceforth, the contextual and implementation influencing factors, both barriers and facilitators, will be collectively referred to as determinants.

## Methods

### Study design

This co-development study utilised a collaborative co-development approach, informed by Community-Based Participatory Research (CBPR), involving individuals with lived or professional experience, informed by prior recommendations and guiding principles [36]. Ethical approval was obtained from the Sport and Exercise Science Research Institute (SESRI) Research Ethics Filter Committee at Ulster University, in Northern Ireland (SESRI-24-056-A).

### Researcher positionality

Throughout the co-development process the research team employed a relative ontological stance, which recognises reality as individually constructed and subjectively experienced [37]. This positionality ensured that the research reflected multiple perspectives and was inclusive of participants’ lived experiences of promoting and supporting physical activity.

### Participants and recruitment

Two workshops were hosted with a mix of parent representatives, SEN school staff (including teachers, classroom assistants and leadership team), representatives from the Education Authority Northern Ireland (EANI) and professionals from regional community sports clubs and national governing bodies (such as Special Olympics Ireland). The aims of the workshops were to explore contextual determinants, resource needs, training requirements, implementation factors, and outcome indicators for adopting and maintaining the fidelity of the WSA to physical activity in SEN settings.

Recruitment was conducted remotely by the research team with assistance from schools and community groups. Recruitment began 05/10/ 2024 and completed prior to first workshop on 29/11/2024. Principals within schools and gatekeepers at target organisations were contacted initially for approval to recruit participants from their organisation. Each collaborating organisation or school was asked to circulate an invitation to participate along with participant information sheets and consent forms. The aim was to recruit at least two participants per participant category (parent/carer, school staff, advocacy/support organisations) through purposive sampling to ensure a diverse range of professional roles or lived experience [38], which was achieved in the recruitment approach. Recruitment of schools adopted a purposive approach with 39 special schools from across Northern Ireland initially contacted. The aim was to ensure representation of schools from across the region. The final six participating schools represented five of the 11 local council areas, spanning from the east of the region to the northwest. The schools were primarily located in urban and suburban areas rather than rural areas, reflecting current patterns of special school provision in Northern Ireland, as these locations have historically provided the necessary population base and specialist infrastructure to support dedicated special education provision.

Participants were recruited if they met one of the following criteria: 1) Parent or primary caregiver of child or young person aged 5–18 years old attending a SEN school in Northern Ireland; 2) Teacher, school leadership or other school staff member of a SEN school setting in Northern Ireland; 3) Professional working with a community or support organisation for families of children or young people with SEN. Prospective participants were given the opportunity to discuss their participation with the research team via telephone and email.

Following consultation with all potential participants via email and telephone, those who met the inclusion criteria were invited to participate in the co-development workshops. Participants were reimbursed for their time participating in and travel to the workshops, per INVOLVE guidelines [39].

### Co-development workshops

The two in-person workshops took place at two university campus locations across Northern Ireland to encourage people from across the region to take part. Each workshop lasted approximately four hours. Discussions across the workshops were guided by the theoretical frameworks underpinning this study and supported the analysis of the qualitative data for population of the IRLM. To support participant’s understanding and engagement in discussions, implementation terms and concepts were defined using lay language, with relevant examples provided to illustrate their meaning and application during each workshop activity.

Workshop 1 explored the group’s thoughts and experiences with regards to supporting physical activity in SEN settings and explored the determinants of physical activity within SEN settings. Findings from our prior work around implementation of physical activity interventions for children and young people with intellectual and developmental disabilities informed the initial discussions during Workshop 1 [40]. The exploration of determinants was guided by the CFIR 2.0 determinants framework, and participants were additionally asked to consider perspectives across the SEM, considering pupils, staff, school and wider school community (Objective 1). Following this, discussions focussed on scoping current strategies used in schools to address barriers and leverage facilitators, informed by the ERIC implementation strategies taxonomy (Objective 2). Workshop 2 explored an assessment of current provision of physical activity in SEN settings, school physical activity policies, and the monitoring and evaluation of physical activity in SEN settings. Outcomes were considered aligned with the IOF framework, with COM-B and the NPT informing initial considerations of the mechanisms underlying those outcomes (Objective 3). The workshop process and subsequent analysis embedded with informing theoretical frameworks is outlined in Fig 1.

**Fig 1:**
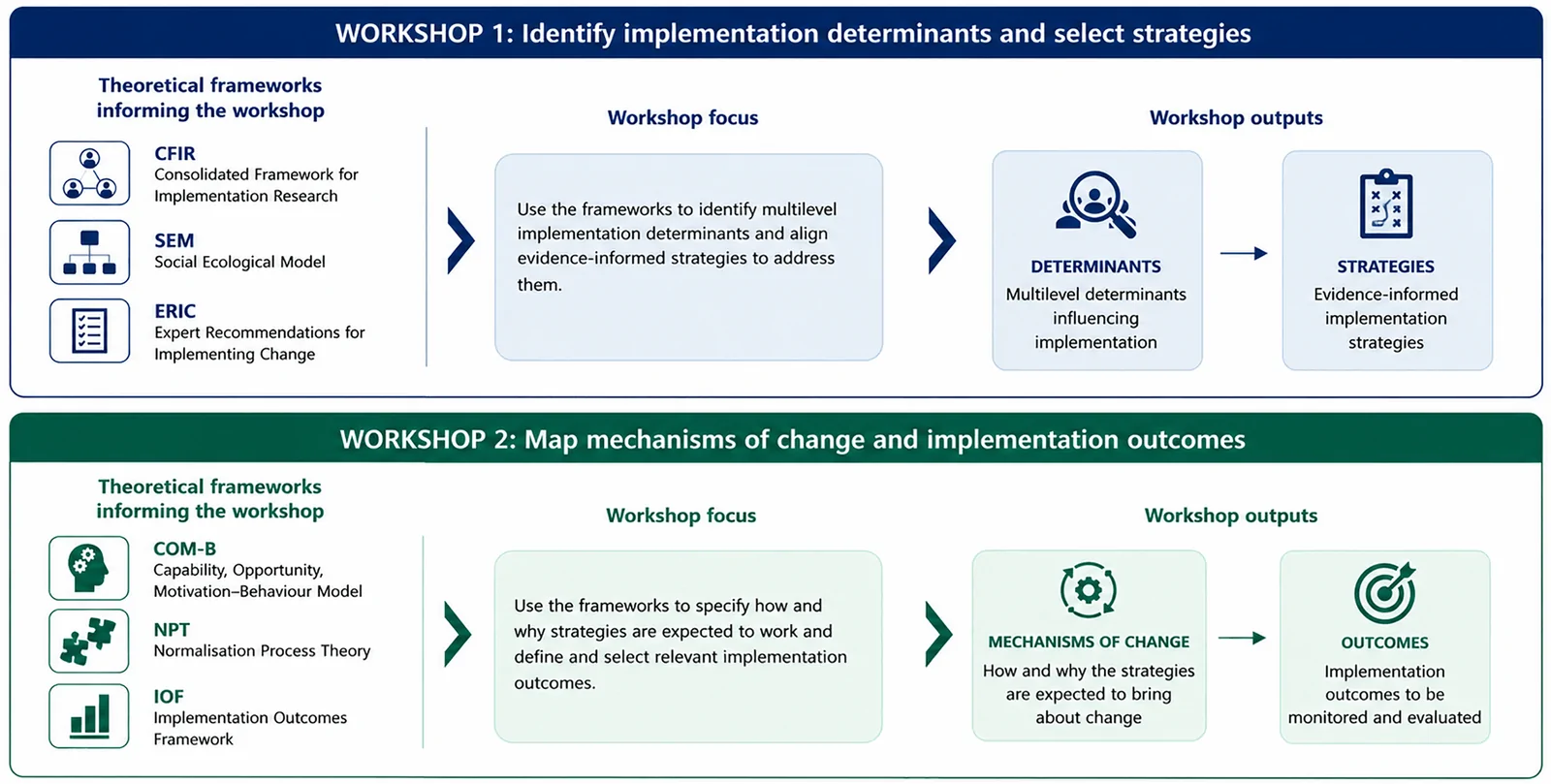
Process flow for mapping of Implementation Research Logic Model.

Upon completion of both workshops, outcomes were fed back to the participants, to solicit feedback on the accuracy of identified determinants, strategies and outcomes (short/long-term) before being further refined.

## Data analysis

Workshop discussions were recorded using an Olympus DM720 digital recorder [41] and transcribed using Microsoft Word audio file transcription with human review. Data was analysed qualitatively alongside flipchart mind maps developed during discussions and through fieldnotes recorded by the research team, using deductive content analysis [42]. The transcripts and data files were read and coded according to the pre-defined domains within the behavioural theory (COM-B) and implementation theories, models, and frameworks (CIFR 2.0, ERIC, IOF and NPT), and the IRLM. Initial coding was conducted through a summarisation of all discussion transcripts and other data files, with grouping of similar response content. The coding was conducted together by two members of the research team (AB and EB), with all disagreements resolved through discussion. The wider research team then met to discuss and refine these response categories.

### Objective 1: Mapping of determinants to CFIR 2.0

The lead author (EB) first reviewed the overarching categories of determinants in the summary document; thereafter, the categories of determinants were mapped to the CFIR 2.0 domains of Innovation, Outer Setting, Inner Setting, Individual Characteristics and Process, based on similarity or applicability by reviewing the names and definitions of the CFIR constructs. Two researchers (JW and LT) conducted a check to ensure that the determinants had been mapped appropriately to CFIR 2.0 constructs resulting in consensus among the team.

### Objective 2: Identifying implementation strategies

To determine appropriate implementation strategies to address the identified determinants highlighted in workshop discussions, the lead author (EB) used CFIR‐ ERIC Implementation Strategy Matching Tool (hereinafter ‘CFIR‐ERIC tool’) developed by Waltz et al. [26]. The CFIR–ERIC tool was developed through a concept mapping and expert consensus process led by implementation science researchers. The matching tool provides a matching matrix showing the strength of association between specific CFIR determinants and ERIC strategies. Using the CFIR‐ERIC tool within this study allowed the research team to input the identified CFIR based determinants into the tool, generating a ranked list of ERIC implementation strategies most likely to address those specific determinants. Identified strategies are prioritised as either Level 1 (strategies endorsed by ≥50% of implementation science experts defined by Waltz et al. [26]) or Level 2 (endorsed by 20% to 49.9% of experts) [36]. The output was then analysed to understand the patterns of expert-endorsed implementation strategies to be used to address or leverage the CFIR determinants from workshop discussions, allowing the tailoring of these strategies to the context of this current study.

### Objective 3 and 4: Building the IRLM

After the identification of the determinants and implementation strategies, these items were mapped into an IRLM [35 (p158)]. Implementation outcomes identified in workshop discussions were deductively coded using the Implementation Outcomes Framework (IOF) [24]. Definitions of implementation outcomes based on the IOF were reviewed, and outcomes identified from the workshop discussions were subsequently divided into implementation outcomes, school-level outcomes, and pupil-level outcomes. The frequency of coded references to each implementation outcome was subsequently calculated to indicate the relative prominence of each outcome within the workshop discussions but should not be interpreted as measures of their relative importance. Finally, through discussion among the research team and review of the qualitative data from workshops, we provided a rationale (causal mechanism) for how the developed strategies worked to address determinants and contribute to specific implementation outcomes. This systematic approach provided a transparent representation of the causal pathway underpinning the implementation of a WSA to physical activity in SEN settings. Mechanisms were informed by the COM-B model of behaviour change for strategies that influenced individual level outcomes [28,30], and the NPT [27,29] for strategies that influenced school-level outcomes.

## Results

Sixteen participants (male = 6, female = 10) with lived or professional experience in special education participated in both workshops consisting of parents (n = 2), teachers (n = 4), classroom assistants (n = 1), school leadership team (n = 2), representatives from the EANI (n = 5) and professionals from community groups and voluntary sector bodies (n = 2).

### Determinants of a whole school approach to physical activity

Guided by CFIR 2.0, we identified 31 determinants of implementation for WSA to physical activity in SEN settings. All determinants were successfully mapped to CFIR construct domains. These include Innovation factors (n = 2), Outer Setting factors (n = 5), Inner Setting factors (n = 11), Individual factors (n = 4), and Process factors (n = 9). Within these, 18 facilitators and 12 barriers to implementing WSA to physical activity in SEN settings were identified, with one item identified as both barrier and facilitator (Fig 2).

**Fig 2:**
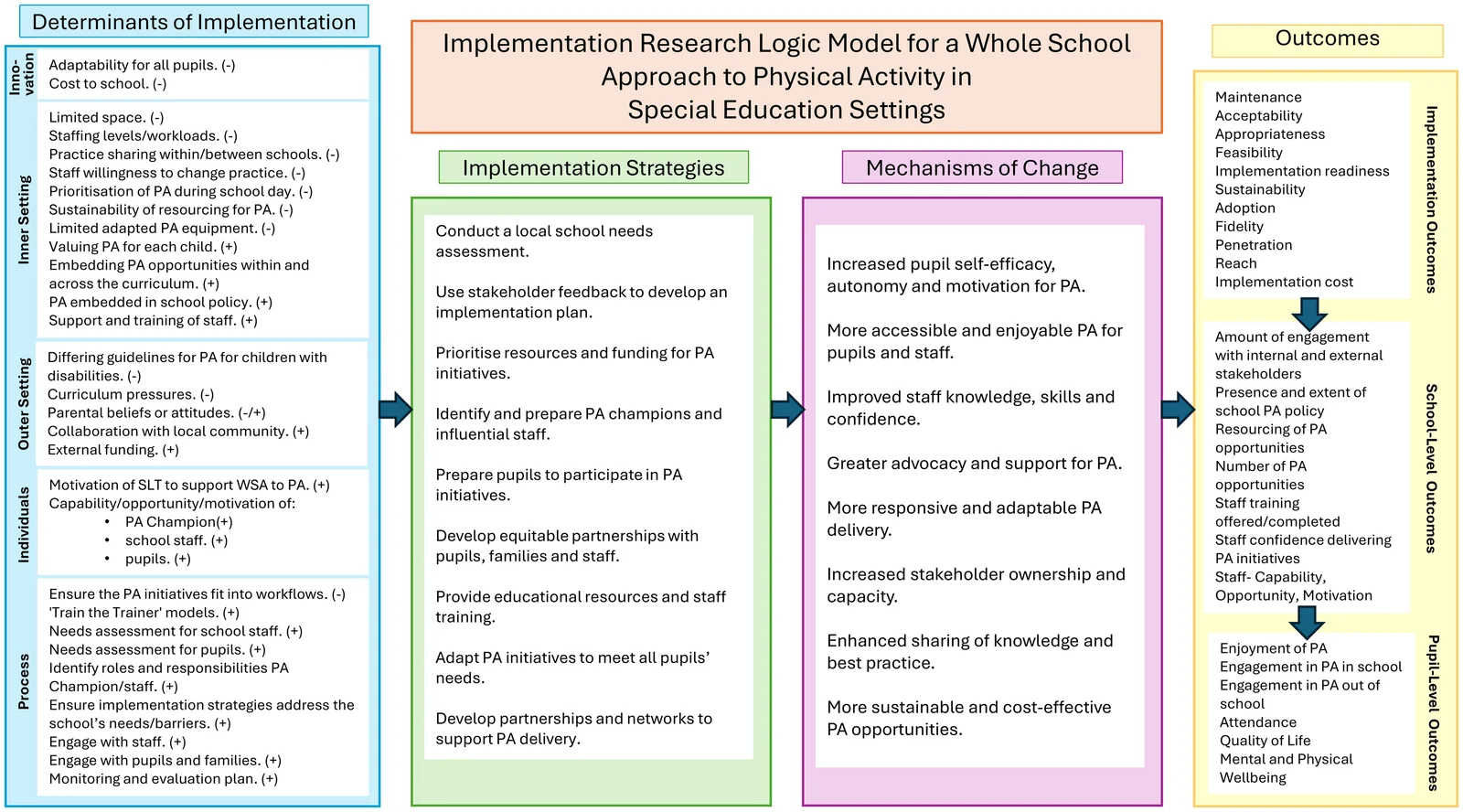
Implementation Research Logic Model for a Whole School Approach to Physical Activity in Special Education Settings.

### ERIC implementation strategies

Overall, the CFIR-ERIC Matching Tool outputted 18 Level 1 strategies and an additional 49 Level 2 strategies. These strategies represented seven of the core conceptual strategy areas (Adapt and tailor to context; Develop stakeholder interrelationships; Engage consumers; Provide interactive assistance; Train and educate stakeholders; Use evaluative and iterative strategies; Utilise financial strategies). S1 Table provides detailed information on which ERIC strategies align to which CFIR 2.0 determinant. The Level 1 strategies identified were summarised into eight pragmatic implementation strategies for SEN settings to employ when implementing a WSA to physical activity (Fig 2).

### Mechanisms

Eight causal mechanisms were developed to explain the pathway between the eight pragmatic implementation strategies, through to the individual level, school level, and implementation outcomes that may be influenced by that strategy (**Table 1**).

**Table 1:** Mechanistic model: Eight pragmatic strategies influencing outcomes at individual, school, and implementation levels.

Level 1 ERIC Strategies	Pragmatic Strategy	Mechanism of Change	Example Related Outcomes
Promote adaptability	Promote adaptability of PA initiatives to meet needs of all pupils.	Physical activity participation becomes more suitable, easier and more enjoyable for children to take part in and for teaching staff to deliver	Enjoyment of PA, Engagement in PA in school, Engagement in PA out of school, Staff confidence delivering PA initiatives, Acceptability, Appropriateness
Build a coalition Develop academic partnerships Promote network weaving	Develop partnerships and promote network weaving with other schools, academic institutions, healthcare providers and community providers to deliver the PA initiatives.	Knowledge, resources, and opportunities can be shared between colleagues and networks and ensure that best practice can be duplicated and implemented across schools	Number of PA opportunities, Staff- Capability, Opportunity, Motivation, Maintenance, Sustainability, Fidelity
Identify and prepare champions Inform local opinion leaders	Identify and prepare PA champions and engage with influential staff members.	Champions and influential staff advocate for PA opportunities increasing school community understanding of the benefits and value of PA, motivation and confidence implementing PA initiatives across the school day for all pupils	Staff confidence delivering PA initiatives, Staff- Capability, Opportunity, Motivation, Implementation readiness, Sustainability
Prepare pupils to be active participants	Intervene with and prepare pupils with additional needs to be active participants in PA initiatives, to enhance uptake & adherence.	Pupils will have increased self-efficacy, autonomy and motivation to engage in PA initiatives within school setting	Enjoyment of PA, Engagement in PA in school, Engagement in PA out of school
Involve pupils and family members Intervene with pupils to enhance uptake & adherence	Involve pupils, family and staff in PA initiatives- develop equitable partnerships between stakeholders.	Staff, school and family feel a sense of ownership in the PA initiatives and capacity to provide PA opportunities for pupils will be increased	Amount of engagement with internal and external stakeholders, Resourcing of PA opportunities, Number of PA opportunities, Staff- Capability, Opportunity, Motivation, Penetration, Reach
Conduct educational meetings Develop educational materials Distribute educational materials	Develop educational materials and conduct training for staff delivering PA initiatives, including classroom support staff.	Training programmes for teachers and staff will ensure everyone has the necessary skills and knowledge to enhance PA opportunities within their school. New training programmes with defined content will ensure accountability, self-efficacy, and confidence.	Staff training offered/completed, Staff confidence delivering PA initiatives, Staff- Capability, Opportunity, Motivation, Implementation readiness
Conduct local needs assessment Obtain and use pupils and family feedback Develop a formal implementation blueprint Facilitation	Conduct local needs assessment of school- Obtain and use staff, pupil, and family feedback and develop a formal implementation plan.	School and staff will be responsive to the needs of pupils, modifying work patterns and practices in response to continued appraisal of PA interventions and their delivery.	Amount of engagement with internal and external stakeholders, Sustainability, Maintenance, Adoption
Access new funding Alter incentive/allowance structures	Access and prioritise resourcing and funding for PA initiatives.	PA initiatives become a cost-effective and sustainable option to enhance the wellbeing of pupils	Quality of Life, Mental and Physical Wellbeing, Resourcing of PA opportunities, Presence and extent of school PA policy, Implementation cost

Abbreviations: PA Physical activity.

### Implementation outcomes

Eleven implementation outcomes, seven school-level outcomes, and six pupil-level outcomes were identified. Fig 2 presents the identified outcomes and the frequency with which each outcome was mentioned across the workshop discussions. The most frequently identified implementation outcomes were reach (n = 11), acceptability (n = 9), and sustainability (n = 4), whereas adoption (n = 1) was identified least frequently. The most frequently identified school-level outcomes were amount of engagement with internal and external stakeholders (n = 6), staff training offered/completed (n = 5) and resourcing of physical activity opportunities in school (n = 5), whereas staff confidence delivering physical activity initiatives (n = 2) was identified least frequently. The most frequently identified pupil-level outcomes were pupil enjoyment of physical activity (n = 10), engagement in physical activity in and out of school (both n = 5), whereas attendance (n = 2) was identified least frequently (Fig 2).

### Implementation research logic model

The co-developed and refined IRLM is presented in Fig 2. This IRLM attempts to link implementation determinants to strategies, calling out the mechanisms through which they will work and identifying which outcomes are targeted (i.e., implementation outcomes, individual and school-level outcomes).

For example, the support and training of staff was identified as a barrier to implementing WSA to physical activity in SEN settings within the inner setting domain of the CFIR 2.0. Conducting training for staff delivering the physical activity initiatives, including classroom support staff, was identified as a Level 1 recommendation from the CFIR-ERIC tool. It was proposed that these training programmes for teachers and staff would ensure everyone has the necessary skills and knowledge to enhance physical activity opportunities within their school (causal mechanism), leading to increased staff confidence delivering physical activity initiatives (outcome). This causal mechanism is based on the COM-B model of behaviour change, specifically using targeted training strategies to build the cognitive and psychological capability of teachers and classroom assistants to deliver the physical activity initiatives.

## Discussion

This study is the first of its kind to explore the implementation of WSA to physical activity specifically within special education settings. Through hosting of co-development workshops, we were able to populate an IRLM, that has informed the development of guidance to support special education providers to implement a WSA to physical activity [43]. The IRLM developed links the implementation determinants to strategies, calling out the mechanisms through which they are proposed to work and identifies which outcomes (implementation outcomes, individual and school-level outcomes) are targeted. Therefore, this IRLM can work as a blueprint for schools to have an overview of what needs to happen, explain how and why the WSA to physical activity is implemented, guide the selection of implementation strategies, inform a monitoring and evaluation plan, and identify areas where adaptations are needed.

### Implementation determinants

Work by Jones, Longbon, and Williams [44] and Craig et al. [45] identified several determinants of mainstream school-based physical activity interventions similar to this current study, including staff confidence, competing priorities, traditional models of teaching, external pressures from regulatory bodies, such as Ofsted, leadership values, and having dedicated physical activity leads. Similarly, Tibbetts et al. [46] explored barriers to WSA to physical activity in mainstream school settings at both the individual level and school level. As with our study, factors such as school culture, staff capacity and workload, school infrastructure and finances, and school priorities all influenced outcomes of a WSA to physical activity.

Discussions with workshop participants highlighted several contextual challenges specific to special education settings when implementing physical activity interventions, emphasising the need to consider these environments separately from mainstream education settings. In particular, participants identified additional requirements, including access to specialist adaptive physical activity equipment, increased 1:1 support for pupils with severe learning or intellectual disabilities and complex needs, and flexible, adaptable initiatives that can accommodate the diverse needs of all pupils. Schools may address these challenges through individualised planning, collaboration with specialist staff and external organisations, creating safe physical activity environments and purposeful adaptation of activities to ensure that pupils with varying abilities and support needs can participate meaningfully [47]. These findings emphasise that successful implementation in special education settings requires consideration of both the intervention itself and the wider organisational and resource-related context in which it is delivered.

### Implementation strategies

School staff, particularly teachers, have been identified as one of the main influencers for promoting physical activity in schools for children with intellectual disabilities [48]. As a result, there is a need to ensure adequate teacher training to support inclusive physical activity and Physical Education (PE) opportunities for children with additional needs and disabilities, through both initial teacher training and continuous ‘in-service’ training [49]. Training and educating teachers, school staff, and families is a vital step in implementing a WSA to physical activity as it fosters buy-in and support from staff and leadership, promotes collective responsibility, enhances staff motivation and autonomy, and ensures a collaborative learning environment that promotes high-quality play and physical activity opportunities [50]. Staff and family education should include developing an awareness of the benefits of physical activity and current physical activity recommendations, increasing confidence to implement physical activity across the school day, promoting the generation of ideas for specific programmes or approaches to implement within the school setting, and allowing for the sharing of experiences [51].

Another key implementation strategy identified for the WSA to physical activity is to identify and prepare Physical Activity Champion(s) within the school. The Physical Activity Champion is often responsible for general oversight and co-ordination of physical activity programmes and initiatives in the school, leading the WSA to physical activity implementation team, advocating for change, and assessing the school’s needs, resources, environment, policies and staff attitudes regarding physical activity promotion [52]. For many schools, the most logical person identified to serve as Physical Activity Champion was the PE teacher, however, this might not be feasible for all special education settings [53]. Studies have shown that non-PE specialist teacher champions may have an advantage within this role as they are more aware of the challenges and priorities faced in the classroom setting, and therefore potentially have more influence for embedding physical activity approaches across the school [54,55]. Additional training for Physical Activity Champions is warranted and should include developing leadership and practical skills to engage stakeholders, advocate for and facilitate change, and implement whole-school physical activity and active play strategies [56].

To promote and maintain physical activity, movement and active play in special education settings building a collaborative network was identified as a key strategy. Engaging with the wider community, including families, community organisations and other schools, has been identified as a key pillar within WSA and can enhance pupil movement behaviours within the school and beyond [17,57]. A scoping review by Michael and colleagues [58], identified six implementation strategies that school- based interventions used to engage families, community members and organisations in physical activity initiatives that positively impacted pupil physical activity behaviours. These implementation strategies included communication with families and community members, education classes and support for families, volunteer opportunities for families and community members, involvement of families and community members in decision making, reinforcement of health knowledge and practices in the home and community environment, and collaborations among school and community to support pupils and their families. By considering these implementation strategies, schools can explore a range of approaches to engage families and the community to support WSA to physical activity in SEN settings.

### Mechanisms of change

Understanding *how* implementation strategies produce change remains a major challenge, especially when trying to scale effective programmes. Real-world implementation unfolds in complex systems, and implementation strategies or bundles used can vary widely even within a single implementation plan [59]. This diversity, layered onto complex contexts, makes it difficult to pinpoint exactly how the utilised strategies influence implementation outcomes. Without clear articulation of strategy components and underlying mechanisms, such as in the developed IRLM, researchers and practitioners will continue to struggle to select, accept, adapt, and scale strategies that reliably achieve intended outcomes [60]. Although tools such as IRLM offer an opportunity for implementers and researchers to describe mechanisms, they provide limited step-by-step guidance on how to approach this task.

For this study we chose two different change-theory models, to guide the development of these mechanisms of change; the COM-B model and the NPT. The COM-B was chosen to explain the mechanism of change due to implementation strategies that address individual-level behaviour (pupil, family and school staff). This theoretical model was chosen due to its current integration into the updated CFIR framework [25], as well as its applicability for implementation strategy development and evaluation [61]. It has been used in other research that involved developing IRLM in WSA to physical activity research [30] and allowed exploration of how specific strategies influenced the capability, opportunity and motivation of individual stakeholders involved in the WSA to physical activity and how these led to specific individual level behaviour outcomes.

The NPT helps explain the cognitive and social work that individuals and groups undertake to integrate and sustain an innovation or intervention in routine practice. Building on earlier empirical work on how new practices become normalised, the NPT organises this into four core generative mechanisms; Coherence, Cognitive Participation, Collective Action, and Reflexive Monitoring [62]. These help to explain how people make sense of an innovation, mobilise others, enact new ways of working, and appraise their ongoing use [29]. We utilised the NPT to develop mechanisms of change within the resultant IRLM to provide a clear, theory-informed rationale for how whole-school targeted implementation strategies may produce desired outcomes. It helps organisations and practitioners identify the processes through which key stakeholders understand the WSA to physical activity, organise themselves to deliver it, operationalise new practices, and evaluate their impact over time. When used alongside frameworks such as CFIR, ERIC and the IOF, the NPT offers a complementary lens for examining the dynamic interplay between contextual determinants and the social and cognitive processes that shape implementation success [29].

### Applying implementation science frameworks in special education settings

The application of implementation science frameworks to the co-development of a WSA to physical activity in special education settings provided several insights beyond those anticipated when the frameworks were selected. Collectively, these implementation science theories, models, and frameworks offered a useful structure for identifying and considering the multiple and interconnected factors influencing physical activity implementation across the school environment. However, their application also highlighted that implementing a WSA in SEN settings cannot be understood solely through individual determinants, strategies, or outcomes. Like findings in other school-based implementation studies [63], participants’ discussions emphasised the interconnected nature of school-, staff-, pupil-, and family-level factors, with the needs and experiences of pupils often influencing how implementation strategies could be perceived and applied across the wider school context. This highlighted the importance of considering the unique characteristics of special education settings, including the diversity of pupils’ abilities, needs, and preferences, alongside organisational factors and the wider school environment.

Our experience also demonstrated that the frameworks were most valuable when used as complementary and flexible guides rather than as prescriptive approaches. While they supported a systematic consideration of implementation and helped to ensure that important factors were not overlooked, participants’ lived experiences and priorities did not always fit neatly within predefined constructs or pathways. Applying the frameworks throughout the co-development process therefore required an iterative approach, in which implementation concepts were informed and, where necessary, adapted according to stakeholder perspectives. Prior to undertaking this study, we anticipated that the frameworks would primarily provide a structured method for organising the co-development process [61]. However, we learned that their greatest value was in facilitating a more comprehensive understanding of the complexity of implementing a WSA to physical activity in SEN settings. This suggests that implementation science frameworks may be particularly useful for guiding WSAs when applied in combination, and iteratively, with stakeholder experience used to contextualise and shape their application [64].

### Strengths and limitations

An important strength of this study was the recruitment and inclusion of participants in the workshops from a diverse range of professional and lived experience backgrounds in special education. Since participants who self-selected for participation in the co-development workshops may be more invested in enhancing physical activity of children with additional needs and disabilities, a targeted recruitment strategy was used to invite participants from various roles to reduce selection bias (e.g., senior leadership teams, classroom assistants, PE and non-PE specialist teachers). A decision was made prior to the study to keep the workshop participant numbers relatively small to aid in depth of discussion and quality of output. This smaller sample size, however, impacted participant numbers within each stakeholder group. As a result, some implementation determinants and strategies relevant to these stakeholder groups may not have been fully captured. Additionally, although the workshops were designed to encourage equitable participation, through structured workshop activities and facilitation, differences in stakeholder roles and perceived authority may have influenced discussion dynamics. In particular, perceived power imbalances between professional stakeholders and parents may have affected the extent to which some participants contributed.

A limitation of this study is the specificity of the findings to the Northern Irish special education context. Models of special education vary around the world, and therefore direct application of the findings of this study and the developed IRLM to special education settings outside of Northern Ireland should be treated cautiously. However, many of the determinants identified in this study are likely to resonate with special education populations worldwide, making this study a valuable source of insight and learning. Consequently, the IRLM should be viewed as a flexible framework rather than a prescriptive model. Researchers and practitioners in other education systems or contexts are encouraged to use the model as a starting point, engaging local stakeholders to validate and adapt its components, ensuring alignment with their specific context and implementation priorities. Future research should examine the transferability and adaptation of the IRLM across diverse special education settings internationally.

Finally, due to the scope of this current study, we were unable to include pupils in the co-development workshops to ensure their voices were heard, specifically in relation to the determinants of implementing WSA to physical activity in SEN settings. However, pupils within special schools in Northern Ireland have taken part in Patient and Public Involvement work throughout the larger project and were instrumental in concept development and informing initial discussions within this project. Additionally, due to the more central role of parents as advocates for this population, the inclusion of parent representatives in the workshops ensured that pupil experiences were considered. However, we acknowledge the limitations in this, even for children with more severe or profound intellectual disabilities and are cautious about assuming the validity of views passed on via parents and caregivers as a proxy voice for their child [65].

Future research should focus on initial feasibility testing and trialling the efficacy of the WSA and IRLM and the identified strategies using hybrid effectiveness trials [66] and evaluating the utility of the developed guidance document to support schools in implementing WSA to physical activity in SEN settings.

## Conclusion

Through this novel study, we have been able to co-develop an IRLM using principles of implementation science and behavioural science to support the implementation of WSA to physical activity in SEN settings. The proposed model illustrates the interactions and causal mechanisms between implementation determinants and implementation strategies to achieve the intended outcomes. This IRLM has formed the basis of a co-developed comprehensive guidance document for implementing WSA to physical activity tailored to the needs of SEN settings, addressing the unique challenges faced in promoting physical activity among pupils with additional needs and disabilities. This newly developed IRLM and guidance document will assist researchers and implementing school partners in designing, delivering, reporting and evaluating the implementation of WSA to physical activity in SEN settings.

## Supporting information

S1 Table CFIR ERIC Matching Tool Output. (XLSX)

S1 Dataset Coded study dataset CFIR_ERIC_IOF. (ZIP)
