# Supplementary material for: Embedding behavioural and implementation science in a whole school approach to promote physical activity among children and young people with disabilities
Source: PLoS One. 2026 Nov 13;21(11):e0362562. doi: 10.1371/journal.pone.0362562 (PMC13766267; doi:10.1371/journal.pone.0362562)
Supplement: Supplementary file 2 [file pone.0362562.s003.docx]

Title: Embedding Behavioural and Implementation Science in a Whole School Approach to

Promote Physical Activity Among Children and Young People with Disabilities

Overall Statement: Thank you for the opportunity to review this manuscript. This manuscript aimed to develop an implementation research logic model to guide the adoption of a whole school approach to physical activity within special educational needs settings. The community-based participatory approach, involving parents, teachers, and other community stakeholders throughout the formulation, strengthens the IRLM’s relevance and potential for real-world impact. I believe the findings can have a profound impact on school-based physical activity promotion, particularly among children and young people living with disabilities. I have provided detailed, section-by-section comments for the authors that I believe will strengthen the paper.

Abstract

- The number of barriers and facilitators identified from workshop discussions does not seem to add up to the number of implementation determinants
- Similar to a previous comment, the authors state that 8 strategies were developed but only list 3. If you are using these as an example they should be placed in parentheses (e.g., staff training, building collaborative networks, engaging families).

Background

- There appears to be an extra space between lines 72-73 between sentences 2 & 3 that should be removed.
- The introduction to Whole of School Approaches is good, but Lines 71-72 stick out from the rest of the paragraph. These lines would be better suited for inclusion in paragraph 3 (lines 81-95), or as the transition sentence at the end of paragraph 2.
- The subheading for Paragraph 3 (lines 81-95) does not align with the text. Most of Paragraph 3 provides information that overlaps and/or compliments paragraph 2 (Overview of what a Whole of School Approach is). There needs to be much more than 1 sentence that discusses what’s been done in schools as it pertains to physical activity and special education/needs
- Lines 106-108, particularly “considering the unique contextual needs,” should be elaborated on; otherwise, the reader is left assuming what these are
- Line 113: it is difficult to identify which factors contributed to successful adoption and implementation, as a WSA is not only adopted but implemented too. The authors state implementing earlier in the sentence but it should be included in both places
- Delete comma in line 128
- Line 135-136 seems to be missing words or has a misplaced comma.
- Lines 138-141 should be simplified further. The aim currently is too wordy and is not communicated effectively enough.
- Making Objective 1 more concise would improve clarity; the remaining objectives are well-written

Methods

- Line 249 about purposive sampling should be introduced 1-2 paragraphs earlier in the recruitment section as it conflicts with the previous line about those who were already invited to participate
- The authors state the workshops were guided by the IRLM. Was there anything done to help parents/guardians understand implementation science and theories/frameworks?
- It is unclear who did the initial coding – was this AB & EB or someone else? Did the coders code independently or together? Did the coders ever meet to check the consistency in their codes? How were differences in codes resolved?
- Line 293-294: were all determinants successfully mapped to CFIR or was this the categories of determinants that were grouped together? Also, the final sentence reads like Results rather than Methods.
- Line 307: there are many things being referred to as determinants (contextual and implementation factors, barriers and facilitators). I recommend greater consistency throughout the paper

Results

- The barrier and facilitator counts add up to 32 determinants, not 31. Is there one missing?
- Organizing the example outcomes by level (individual, school, etc.) within the far-right column would help the reader understand the level at which these are operating
- Line 351-353: Move this to Methods
- There is a lack of information provided about the implementation outcomes. It was difficult to see the text in the figures provided, but since these were deductively coded, information on how frequently these outcomes were mentioned should be available. This is something that could be reported to strengthen the Results
- This section flips back and forth between active and passive voice
- How many people attended each workshop?

Discussion

- Line 385 is too vague (“several unique contextual challenges”). Since it was not provided in the first paragraph of the discuss, it is recommended that the authors provide an overview/recap of what those unique challenges. This will help the reader understand what they are compared later in the paragraph when an overview of existing literature is presented.
- Recent work by Craig et al. (<https://doi.org/10.1186/s12966-025-01815-7> & <https://doi.org/10.1186/s12889-024-17744-2>) has also found some similar results when studying implementation of a whole of school approach, albeit lacking a focus on the special needs context.
- The extra space between lines 388 and 389 should be removed.
- Lines 397-391: This is good, but could be better connected with the paragraph above. Another option is to include how schools address these factors. The current structure of the discussion with subheadings actually makes the narrative read less connected. In its current form, this section of text is just a rewrite of the results.
- Lines 413-417: One sentence should not be a standalone paragraph. This should follow a similar structure as the paragraph immediately before and after.
- Lines 425-427: Citations are needed
- Limitations: The small sample is a major limitation. In particular, there were very few parents represented (n=2) and a limited amount of those who work in schools every day (n=4), and who will be in charge of implementing interventions, that participated.
- Limitations: There was no mention of who attended each workshop and whether they were balanced. Power dynamics between EANI reps and parents could have been present and may have influenced who spoke, how often, and what the responses were.
- Limitations: How many schools were represented? There were 4 teachers, 1 classroom assistant, and 2 from school leadership teams, but were all of these from different schools?

Other

- The text within the figures was extremely blurry in multiple places, including all of Fig 2, and could not be read
