# Supplementary material for: Embedding behavioural and implementation science in a whole school approach to promote physical activity among children and young people with disabilities
Source: PLoS One. 2026 Nov 13;21(11):e0362562. doi: 10.1371/journal.pone.0362562 (PMC13766267; doi:10.1371/journal.pone.0362562)
Supplement: Supplementary file 3 [file pone.0362562.s005.docx]

Dear Editor and Reviewers,

We would like to sincerely thank the Editor and the reviewers for the time and effort they devoted to evaluating our manuscript, “Embedding Behavioural and Implementation Science in a Whole School Approach to Promote Physical Activity Among Children and Young People with Disabilities”. We greatly appreciate the thoughtful and constructive feedback provided, which has been invaluable in improving the clarity, quality, and overall contribution of the manuscript. We have carefully considered each comment and have revised the manuscript accordingly. Below, we provide a detailed, point-by-point response to the reviewers’ comments and indicate the corresponding changes made in the marked revised manuscript.

Additionally, I am uploading Figure 2 as both a PDF and TIFF file in hopes that the resolution is improved.

We hope that the revisions and responses provided adequately address the reviewers’ comments and concerns. We sincerely thank the Editor and reviewers again for their careful consideration and constructive feedback, which has helped us to improve the manuscript. We hope that the revised version is now suitable for publication.

Kind Regards,

The Author Team.

| Reviewer Feedback | Response to Reviewer | Location of Amendment |
| --- | --- | --- |
| Reviewer 1 | | |
| Introduction |  |  |
| P. 3, L. 71-72: “However, studies exploring the implementation of WSA in Special Education Needs (SEN) school settings is lacking.” I believe this should be “are lacking” (i.e., studies). | Thank you for this. This correction has now been made. | Line 78-79 |
| P. 5, L. 99-102: I believe that “aids” should be changed to “aid” (i.e., the toolkits referred to). | Thank you for this. This correction has now been made. | Line 109 |
| P. 5, L. 103: for consistency purposes, I suggest sticking to ‘physical activity’, i.e., ‘promote physical activity’ instead of ‘promoting health’, though they are obviously related. | Thank you for this. This correction has now been made. | Line 111 |
| P. 5, L. 118-121: Can you please clarify concisely in what sense the existence of the models and frameworks mentioned below, which, as outlined, appear rather complementary and are used as frameworks underpinning the study, are a significant challenge when applying the whole school approach to implementation planning? | Thank you for identifying this. This has now been clarified at the end of the paragraph. | L134-138 |
| P. 6, L. 143 the abbreviation “CBPR” is mentioned for the first time in the body of the text and I believe should be fully spelled out. | Apologies for this oversight. This has now been clarified in the aims and removed from the study design section. | L246-247 |
| Methods |  |  |
| P. 7, L. 158-160: In my view, and experience, what is presented in this study is a collaborative co-development process (inspired/informed to some degree by the principles of CBPR), but not necessarily a CBPR approach per se, which is usually more encompassing and has more stringent implications (e.g., shared decision making between researchers and end users at key stages of the research process). | Thank you for raising this point. This is now clarified. | Abstract and L225 and L245-247 |
| P. 9, L. 207-209: Can you please clarify concisely how the capability, opportunity, and motivation elements of the COM-B model specify the proximal behavioural mechanisms that drive ‘adoption’? In a related manner, is adoption considered one of ‘specific individual level behaviour outcomes’ that were explored by means of COM-B (p. 20, L. 462-466) in this study? | This has been clarified and an example provided to help reader understanding | L179-182 |
| P. 10, L. 233-240. Recruitment: I understand that qualitative research is not necessarily concerned with ‘representativity’ in the statistical sense of the term. Still, it would be useful to get a sense of how many schools/organisations were approached to participate, how many responded, and the geographical distribution of schools/organisations approached, i.e., were they located across Northern Ireland or in one or a few specific geographical areas? | Thank you for highlighting this. A paragraph clarifying important contextual information for recruitment of special schools from across northern Ireland has been added. | L325-334 |
| P. 11, L. 256-259: It is stated that discussions across the workshops were “guided by the IRLM Framework” (I assumed it is Czosnek et al’s IRLM alluded to in the introduction). Subsequently, it is noted that the study frameworks were used to and support “the analysis of the qualitative data for population of the IRLM”. Am I correct assuming that you are now referring to the IRLM to guide implementation of a WSA to physical activity in SEN settings whose development was the aim of this study? | Thank you for recognising this confusion. Clarification has now been provided. | L351-356 |
| P. 11 L. 254-274: While Figure 1 is a welcome addition, it does not provide enough information on how the workshop process was informed by the theoretical frameworks, and the text provided in the body of the manuscript is not very detailed either. | Figure has been revised and methods amended to clarify | Figure 1 and L363-372 |
| P.12, L. 296-305: The reference number (#28) provided for the ‘CFIR‐ERIC tool’ developed, as noted, by Waltz et al. does not seem to correspond with the actual reference in the reference list corresponding to such number (Leask et al., 2019). Having said this, the information provided in the paragraph about this tool does not allow me to get a clear picture of how this tool works and how it was used in the current study along with its merits. Can you please provide some additional information? For example, are the ‘experts’ mentioned the participants in the workshop? | Thank you for highlighting the referencing error- this is now corrected. Also clarification of the approach is now provided. | L403-415 |
| Results |  |  |
| P. 13. L. 324: please clarify the meaning of ‘non-departmental governing bodies’, which readers from outside the UK may not be familiar with. | This section is clarified for an international reading audience | L440-441 |
| P. 13, L. 326-330: Figure 2 looks like a very useful addition to this section. However, I have been unable to read it properly in the current format. | Figure 2 has now been refined. | Figure 2 |
| P. 14, L. 334-342: As I noted earlier, without having a clearer idea of how the CFIR-ERIC Matching Tool works in practice, it is hard to judge the merits of the information provided here. | Clarified in methods section. | L403-415 |
| P. 16, L. 357-360. As I also mentioned earlier, without being able to read properly Figure 2 it is difficult to judge the merits of the information provided. | Figure 2 has now been refined. | Figure 2 |
| Discussion |  |  |
| Though clearly written and informative, substantial parts of this section look to me like a repetition/extension of the information provided in previous sections about the nature of the frameworks used and why they were used. Personally, I was hoping for greater reflection on their suitability to guide the co-development process according to the project goals and considering the participants’ lived experience and views. For example, what did you learn about using these frameworks that you did not know before undertaking this study? | Thank you for this insightful comment; we have revised the Discussion to include greater critical reflection on the suitability and practical application of the frameworks in co-developing a Whole School Approach to physical activity in special education settings, including what we learned from their use in relation to stakeholder experiences and the need for flexible, context-specific application. | L622-649 |
| P. 21, L.490-494: While I agree with the authors that many of the barriers and facilitators identified in this study are likely to resonate with special education populations worldwide, I also agree with them when they recommend caution regarding application of the findings of this study and the developed IRLM to special education settings outside of Northern Ireland. In this regard, do you have any suggestions to help people outside from Northern Ireland who may be interested in applying the IRLM in their specific contexts? | Thank you for highlighting this omission- We have therefore expanded the limitations/discussion section to clarify that the IRLM is intended as a flexible framework that can be adapted rather than adopted. | L672-678 |
| P. 21, L. 495-500: As part of the appropriate and informative explanation provided to justify why pupils were not included in in the co-development of the IRLM, there is mention to their involvement throughout the ‘larger project’. I believe that providing some concise yet clear information in the Introduction section about the ‘larger project’ will contribute to a better contextualisation of the work presented and its implications. In line with one of my previous comments, it may also help readers see where this study fits within the broader CBPR process alluded to earlier. | Thank you for this helpful suggestion. We agree that providing additional context regarding the larger project will help readers better understand the broader project and how the present study fits within it. We have therefore revised the Introduction to include a brief description of the overarching project | L213-217 |
| P. 21, L. 506-508: Should some initial feasibility testing/trialling be also part of the suggestions for future research briefly outlined here? | Thank you for this suggestion. This is now clarified in L525. | L690 |
| Reviewer 2 | | |
| Introduction |  |  |
| Overall: the introduction is quite long, but I really like it because it explains in detail some key aspects of implementation science which are relevant for the paper. However, I feel that some content of the methods section could add the introduction (CFIR, ERIC, IOF, COM-B, SEM). I encourage the authors to re-think the introduction, and incorporate the descriptive parts of the methods into the introduction. | Amended with the theoretical underpinning incorporated into the introduction | L139-197 |
| Further, please think about adding some kind of a “flow diagram” or figure/overview of the different frameworks and models you utilize, and how they are linked in your approach. Especially readers, that are not that familiar with implementation science will benefit from that. | A redesigned figure is now provided (figure 1) | Fig 1 |
| Methods |  |  |
| Overall: if you explain the frameworks, models,… utilized, I would suggest to always start with the (specific) aim of each method, and then explain how it was utilized. | The use of each framework is now clarified | L363-372 and data analysis subheadings. |
| L233: how many people were approached overall, and also with respect to the three groups? Please add the response rates. Did you have the chance to randomize? | Thank you for highlighting this. A paragraph clarifying important contextual information for recruitment of special schools from across northern Ireland has been added. The concept of randomisation was not applicable in this study as it was not an RCT. | L325-334 |
| Line 238: The wording “The aim was to recruit…” here leads to the question if that aim was achieved or if it was a desired criterion that you did not achieve. | This is clarified to reflect the achievement of this aim for recruitment. | L326-327 |
| Line 248: It was not clear to me whether every participant was required to have a consultation or whether this was voluntary. | This is now amended and clarified. | L342 |
| Line 256-259: As you had 2 workshops with a different focus, you could elaborate on the content of each workshop in the text, not just in the figure. | This has been expanded upon in both the figure and text | L358-383 |
| Line 320-324: You could add who participated in which workshop (distribution groups/ gender). | This is outlined in paragraph 1 of the results section. | L434-438 |
| Strengths and Limitations |  |  |
| Line 489-494: I would suggest providing more background to the specific SEN setting in Northern Ireland here or in the introduction. How is PA typically included in SEN settings? Which SEN settings are you referring to? Schools that only carry to special educational needs? inclusion schools? Providing this information helps readers identify to what extent these findings could be applicable to their context. | Thank you for highlighting this point- the special education context in Northern Ireland has been clarified in the introduction now to hopefully aid in understanding applicability of findings to other settings. | L96-103 |
| I think it is also important to mention earlier that your study only focuses on settings for intellectual and developmental disabilities (as opposed to physical disabilities or mental health challenged children). The first time this is mentioned is in line 245. | As above this is clarified in the introduction section, as Special Schools in Northern Ireland also cater to children with diverse cognitive, sensory and physical profiles. | L96-103 |
| Other |  |  |
| Figure 1: do you really need a figure here? Please consider adding more information to the figure, e.g. some key aspects/tasks etc. that were addressed within each workshop. At the moment, the figure is not working if standing alone. Further, I suggest to order the legend according to the appearance of the frameworks, not in an alphabetical order. | Figure 1 has been refined | Figure 1 |
| Figure 2: I am afraid that figure 2 is too busy, it does not read well if printed or online. | Figure 2 has been refined |  |
| Reviewer 3 | | |
| Abstract |  |  |
| The number of barriers and facilitators identified from workshop discussions does not seem to add up to the number of implementation determinants | This was due to 1 item being identified as both a barrier and facilitator by the group. I hope that we have clarified this point now. | L40-41 |
| Similar to a previous comment, the authors state that 8 strategies were developed but only list 3. If you are using these as an example they should be placed in parentheses (e.g., staff training, building collaborative networks, engaging families). | Thank you for this, these items are now in parentheses. | L43-44 |
| Background |  |  |
| There appears to be an extra space between lines 72-73 between sentences 2 & 3 that should be removed. | Corrected |  |
| The introduction to Whole of School Approaches is good, but Lines 71-72 stick out from the rest of the paragraph. These lines would be better suited for inclusion in paragraph 3 (lines 81-95), or as the transition sentence at the end of paragraph 2. | Thank you for this. This linking sentence has now been moved to the end of paragraph 2. | L78-80 |
| The subheading for Paragraph 3 (lines 81-95) does not align with the text. Most of Paragraph 3 provides information that overlaps and/or compliments paragraph 2 (Overview of what a Whole of School Approach is). There needs to be much more than 1 sentence that discusses what’s been done in schools as it pertains to physical activity and special education/needs | The heading has been reworded, as this section was more about the importance of considering the school’s specific contexts in a WSA and less about evidence on WSA to physical activity in SEN settings (as there is none). | L80 |
| Lines 106-108, particularly “considering the unique contextual needs,” should be elaborated on; otherwise, the reader is left assuming what these are | Thank you for this, this has been elaborated and clarified in the manuscript. | L115-116 |
| Line 113: it is difficult to identify which factors contributed to successful adoption and implementation, as a WSA is not only adopted but implemented too. The authors state implementing earlier in the sentence but it should be included in both places | Thank you for highlighting this. This has now been corrected to add ‘adoption and implementation’. | L121 |
| Delete comma in line 128 | Corrected |  |
| Line 135-136 seems to be missing words or has a misplaced comma. | Thank you for highlighting this. The sentence has been reworked to ensure grammatical correctness. | L205-209 |
| Lines 138-141 should be simplified further. The aim currently is too wordy and is not communicated effectively enough. | Thank you. The primary aim has now been rewritten for clarity. | L211-220 |
| Making Objective 1 more concise would improve clarity; the remaining objectives are well-written | Thank you. Objective 1 has now been rewritten for clarity. | L225-230 |
| Methods |  |  |
| Line 249 about purposive sampling should be introduced 1-2 paragraphs earlier in the recruitment section as it conflicts with the previous line about those who were already invited to participate | This has now been amended. | L325-334 |
| The authors state the workshops were guided by the IRLM. Was there anything done to help parents/guardians understand implementation science and theories/frameworks? | Thank you, this has now been clarified in the methods section. | L354-356 |
| It is unclear who did the initial coding – was this AB & EB or someone else? Did the coders code independently or together? Did the coders ever meet to check the consistency in their codes? How were differences in codes resolved? | This has now been clarified. | L386-388 |
| Line 293-294: were all determinants successfully mapped to CFIR or was this the categories of determinants that were grouped together? Also, the final sentence reads like Results rather than Methods. | This has now been moved and clarified | L442-443 |
| Line 307: there are many things being referred to as determinants (contextual and implementation factors, barriers and facilitators). I recommend greater consistency throughout the paper | Thank you for recognising this. We have defined this more clearly after the study aims. And referred to determinants for the remainder of the paper. | Defined in L238-242 and referred to throughout. |
| Results |  |  |
| The barrier and facilitator counts add up to 32 determinants, not 31. Is there one missing? | This has been clarified. One item was identified to be both a barrier and facilitator. | L448-449 |
| Organizing the example outcomes by level (individual, school, etc.) within the far-right column would help the reader understand the level at which these are operating | This has now been amended in the right hand column with all row outcomes organised from pupil-, to school- to implementation outcomes | Table 1 |
| Line 351-353: Move this to Methods | This has now been moved to the methods section. | L420-422 |
| There is a lack of information provided about the implementation outcomes. It was difficult to see the text in the figures provided, but since these were deductively coded, information on how frequently these outcomes were mentioned should be available. This is something that could be reported to strengthen the Results | Thank you for highlighting this. This has now been amended both in the methods and results section, with the inclusion of frequency counts. | Methods- L418-423  Results- L469-479 |
| This section flips back and forth between active and passive voice | Areas of active voice have now been rewritten in passive voice to ensure consistency | Results section |
| How many people attended each workshop? | All participants attended both session, this has now been clarified | L438 |
| Discussion |  |  |
| Line 385 is too vague (“several unique contextual challenges”). Since it was not provided in the first paragraph of the discuss, it is recommended that the authors provide an overview/recap of what those unique challenges. This will help the reader understand what they are compared later in the paragraph when an overview of existing literature is presented. | I agree that the original structure of this section impacted on clarity. I have now restructured this section to aid in clarity for the reader. | L523-535 |
| Recent work by Craig et al. (<https://doi.org/10.1186/s12966-025-01815-7> & <https://doi.org/10.1186/s12889-024-17744-2>) has also found some similar results when studying implementation of a whole of school approach, albeit lacking a focus on the special needs context. | Thank you for highlighting these references. I have included Craig et al. (2024) as a reference in the discussion section. | L515 |
| The extra space between lines 388 and 389 should be removed. | Thank you for spotting this additional line- this has been corrected. |  |
| Lines 397-391: This is good, but could be better connected with the paragraph above. Another option is to include how schools address these factors. The current structure of the discussion with subheadings actually makes the narrative read less connected. In its current form, this section of text is just a rewrite of the results. | Thank you for highlighting the need to move beyond description here. The paragraph has now been amended to address this. | L529-535 |
| Lines 413-417: One sentence should not be a standalone paragraph. This should follow a similar structure as the paragraph immediately before and after. | Thank you for noting this. I have incorporated the 1 sentence paragraph with the paragraph above. |  |
| Lines 425-427: Citations are needed | I assume that this comment implies that the 1 citation provided is insufficient for the point raised in this section and therefore I have added another citation to support this. (Webster et al, 2015) | L570 |
| Limitations: The small sample is a major limitation. In particular, there were very few parents represented (n=2) and a limited amount of those who work in schools every day (n=4), and who will be in charge of implementing interventions, that participated. | Thank you for highlighting this omission. This has now been amended in the limitations section. | L658-666 |
| Limitations: There was no mention of who attended each workshop and whether they were balanced. Power dynamics between EANI reps and parents could have been present and may have influenced who spoke, how often, and what the responses were. | Thank you for recognising this. We have now amended the limitations to acknowledge that differences in stakeholder roles and experience may have influenced participation during the co-development process. | L662-666 |
| Limitations: How many schools were represented? There were 4 teachers, 1 classroom assistant, and 2 from school leadership teams, but were all of these from different schools? | Thank you for this. As with an earlier point from a reviewer this has now been clarified in the methods section. | L327-331 |
| Other |  |  |
| The text within the figures was extremely blurry in multiple places, including all of Fig 2, and could not be read | Refined | Figure 1 and 2 |
