# Supplementary material for: Embedding behavioural and implementation science in a whole school approach to promote physical activity among children and young people with disabilities
Source: PLoS One. 2026 Nov 13;21(11):e0362562. doi: 10.1371/journal.pone.0362562 (PMC13766267; doi:10.1371/journal.pone.0362562)
Supplement: Peer review history

# Peer review: Decision Letter 0

**DOI:** 10.1371/journal.pone.0362562.r001

**Authors:** Branko Aleksic

24 Mar 2026

Dear Dr. Byrd,

Thank you for submitting your manuscript to PLOS ONE. After careful consideration, we feel that it has merit but does not fully meet PLOS ONE’s publication criteria as it currently stands. Therefore, we invite you to submit a revised version of the manuscript that addresses the points raised during the review process.

Please submit your revised manuscript by May 08 2026 11:59PM. If you will need more time than this to complete your revisions, please reply to this message or contact the journal office at plosone@plos.org. When you're ready to submit your revision, log on to https://www.editorialmanager.com/pone/ and select the 'Submissions Needing Revision' folder to locate your manuscript file.

- A letter that responds to each point raised by the academic editor and reviewer(s). You should upload this letter as a separate file labeled 'Response to Reviewers'.
- A marked-up copy of your manuscript that highlights changes made to the original version. You should upload this as a separate file labeled 'Revised Manuscript with Track Changes'.
- An unmarked version of your revised paper without tracked changes. You should upload this as a separate file labeled 'Manuscript'.

If you would like to make changes to your financial disclosure, please include your updated statement in your cover letter. Guidelines for resubmitting your figure files are available below the reviewer comments at the end of this letter.

If applicable, we recommend that you deposit your laboratory protocols in protocols.io to enhance the reproducibility of your results. Protocols.io assigns your protocol its own identifier (DOI) so that it can be cited independently in the future. For instructions see: https://journals.plos.org/plosone/s/submission-guidelines#loc-laboratory-protocols. Additionally, PLOS ONE offers an option for publishing peer-reviewed Lab Protocol articles, which describe protocols hosted on protocols.io. Read more information on sharing protocols at https://plos.org/protocols?utm_medium=editorial-email&utm_source=authorletters&utm_campaign=protocols.

We look forward to receiving your revised manuscript.

Kind regards,

Branko Aleksic, MD, PhD

Academic Editor

PLOS One

Journal Requirements:

When submitting your revision, we need you to address these additional requirements.

1. Please ensure that your manuscript meets PLOS ONE's style requirements, including those for file naming. The PLOS ONE style templates can be found at

https://journals.plos.org/plosone/s/file?id=wjVg/PLOSOne_formatting_sample_main_body.pdf and

https://journals.plos.org/plosone/s/file?id=ba62/PLOSOne_formatting_sample_title_authors_affiliations.pdf.

2. Thank you for stating the following financial disclosure:

“The co-development work in this study was funded by the Garfield Weston Foundation.”

Please state what role the funders took in the study.  If the funders had no role, please state: "The funders had no role in study design, data collection and analysis, decision to publish, or preparation of the manuscript."

If this statement is not correct you must amend it as needed.

Please include this amended Role of Funder statement in your cover letter; we will change the online submission form on your behalf.

3. We note that your Data Availability Statement is currently as follows: [All relevant data are within the manuscript and its Supporting Information files]

Please confirm at this time whether or not your submission contains all raw data required to replicate the results of your study. Authors must share the “minimal data set” for their submission. PLOS defines the minimal data set to consist of the data required to replicate all study findings reported in the article, as well as related metadata and methods (https://journals.plos.org/plosone/s/data-availability#loc-minimal-data-set-definition).

For example, authors should submit the following data:

- The values behind the means, standard deviations and other measures reported;

- The values used to build graphs;

- The points extracted from images for analysis.

Authors do not need to submit their entire data set if only a portion of the data was used in the reported study.

If your submission does not contain these data, please either upload them as Supporting Information files or deposit them to a stable, public repository and provide us with the relevant URLs, DOIs, or accession numbers. For a list of recommended repositories, please see https://journals.plos.org/plosone/s/recommended-repositories.

If there are ethical or legal restrictions on sharing a de-identified data set, please explain them in detail (e.g., data contain potentially sensitive information, data are owned by a third-party organization, etc.) and who has imposed them (e.g., an ethics committee). Please also provide contact information for a data access committee, ethics committee, or other institutional body to which data requests may be sent. If data are owned by a third party, please indicate how others may request data access.

4. If the reviewer comments include a recommendation to cite specific previously published works, please review and evaluate these publications to determine whether they are relevant and should be cited. There is no requirement to cite these works unless the editor has indicated otherwise.

[Note: HTML markup is below. Please do not edit.]

Reviewers' comments:

Reviewer's Responses to Questions

**Comments to the Author**

1. Is the manuscript technically sound, and do the data support the conclusions?

Reviewer #1: Yes

Reviewer #2: Yes

Reviewer #3: Yes

**********

2. Has the statistical analysis been performed appropriately and rigorously?

Reviewer #1: N/A

Reviewer #2: N/A

Reviewer #3: N/A

**********

3. Have the authors made all data underlying the findings in their manuscript fully available?

The PLOS Data policy

Reviewer #1: Yes

Reviewer #2: Yes

Reviewer #3: Yes

**********

4. Is the manuscript presented in an intelligible fashion and written in standard English?

Reviewer #1: Yes

Reviewer #2: Yes

Reviewer #3: Yes

**********

Reviewer #1: Thanks for the opportunity to review this manuscript. The study presented describes a rigorous, theory-based, process to develop an implementation research logic model (IRLM) for the implementation of a whole school approach (WSA) to enhance the promotion of physical activity in schools for children and young people with additional needs and disabilities. As the authors remark, the newly developed IRLM will assist researchers and implementing school partners in designing, delivering, reporting and evaluating the implementation of WSA to physical activity in special education needs settings. As such, I believe this work shows good potential to contribute meaningfully both to the literature on physical activity promotion for children and young people with additional needs and disabilities and to the implementation science literature. The main issue I see with the manuscript, as currently written, is that it remains very dense from a theoretical/conceptual perspective and it is difficult to see how the views of the the participants in the co-development process contributed to the evolving implementation research logic model. In particular, the manuscript lacks concrete example connecting the theory with what happened during the workshops and subsequent analysis phase. In addition, the manuscript could benefit from clarification and elaboration in some of its sections, as I point out in some of my specific comments below.

Introduction

P. 3, L. 71-72: “However, studies exploring the implementation of WSA in Special Education Needs (SEN) school settings is lacking.” I believe this should be “are lacking” (i.e., studies).

P. 5, L. 99-102: I believe that “aids” should be changed to “aid” (i.e., the toolkits referred to).

P. 5, L. 103: for consistency purposes, I suggest sticking to ‘physical activity’, i.e., ‘promote physical activity’ instead of ‘promoting health’, though they are obviously related.

P. 5, L. 118-121: Can you please clarify concisely in what sense the existence of the models and frameworks mentioned below, which, as outlined, appear rather complementary and are used as frameworks underpinning the study, are a significant challenge when applying the whole school approach to implementation planning?

P. 6, L. 143 the abbreviation “CBPR” is mentioned for the first time in the body of the text and I believe should be fully spelled out.

Methods

P. 7, L. 158-160: In my view, and experience, what is presented in this study is a collaborative co-development process (inspired/informed to some degree by the principles of CBPR), but not necessarily a CBPR approach per se, which is usually more encompassing and has more stringent implications (e.g., shared decision making between researchers and end users at key stages of the research process).

P. 9, L. 207-209: Can you please clarify concisely how the capability, opportunity, and motivation elements of the COM-B model specify the proximal behavioural mechanisms that drive ‘adoption’? In a related manner, is adoption considered one of ‘specific individual level behaviour outcomes’ that were explored by means of COM-B (p. 20, L. 462-466) in this study?

P. 10, L. 233-240. Recruitment: I understand that qualitative research is not necessarily concerned with ‘representativity’ in the statistical sense of the term. Still, it would be useful to get a sense of how many schools/organisations were approached to participate, how many responded, and the geographical distribution of schools/organisations approached, i.e., were they located across Northern Ireland or in one or a few specific geographical areas?

P. 11, L. 256-259: It is stated that discussions across the workshops were “guided by the IRLM Framework” (I assumed it is Czosnek et al’s IRLM alluded to in the introduction). Subsequently, it is noted that the study frameworks were used to and support “the analysis of the qualitative data for population of the IRLM”. Am I correct assuming that you are now referring to the IRLM to guide implementation of a WSA to physical activity in SEN settings whose development was the aim of this study?

P. 11 L. 254-274: While Figure 1 is a welcome addition, it does not provide enough information on how the workshop process was informed by the theoretical frameworks, and the text provided in the body of the manuscript is not very detailed either.

P.12, L. 296-305: The reference number (#28) provided for the ‘CFIR‐ERIC tool’ developed, as noted, by Waltz et al. does not seem to correspond with the actual reference in the reference list corresponding to such number (Leask et al., 2019). Having said this, the information provided in the paragraph about this tool does not allow me to get a clear picture of how this tool works and how it was used in the current study along with its merits. Can you please provide some additional information? For example, are the ‘experts’ mentioned the participants in the workshop?

Results

P. 13. L. 324: please clarify the meaning of ‘non-departmental governing bodies’, which readers from outside the UK may not be familiar with.

P. 13, L. 326-330: Figure 2 looks like a very useful addition to this section. However, I have been unable to read it properly in the current format.

P. 14, L. 334-342: As I noted earlier, without having a clearer idea of how the CFIR-ERIC Matching Tool works in practice, it is hard to judge the merits of the information provided here.

P. 16, L. 357-360. As I also mentioned earlier, without being able to read properly Figure 2 it is difficult to judge the merits of the information provided.

Discussion

Though clearly written and informative, substantial parts of this section look to me like a repetition/extension of the information provided in previous sections about the nature of the frameworks used and why they were used. Personally, I was hoping for greater reflection on their suitability to guide the co-development process according to the project goals and considering the participants’ lived experience and views. For example, what did you learn about using these frameworks that you did not know before undertaking this study?

P. 21, L.490-494: While I agree with the authors that many of the barriers and facilitators identified in this study are likely to resonate with special education populations worldwide, I also agree with them when they recommend caution regarding application of the findings of this study and the developed IRLM to special education settings outside of Northern Ireland. In this regard, do you have any suggestions to help people outside from Northern Ireland who may be interested in applying the IRLM in their specific contexts?

P. 21, L. 495-500: As part of the appropriate and informative explanation provided to justify why pupils were not included in in the co-development of the IRLM, there is mention to their involvement throughout the ‘larger project’. I believe that providing some concise yet clear information in the Introduction section about the ‘larger project’ will contribute to a better contextualisation of the work presented and its implications. In line with one of my previous comments, it may also help readers see where this study fits within the broader CBPR process alluded to earlier.

P. 21, L. 506-508: Should some initial feasibility testing/trialling be also part of the suggestions for future research briefly outlined here?

Reviewer #2: Thank you for the opportunity to review this manuscript. The authors make a valuable contribution to the existing literature by providing an implementation research logic model for whole-school approaches (WSA) to children’s physical activity, based on community-based participatory research. Hereby they consider an often-neglected setting, namely that of special educational needs (SEN). This model can serve as a beneficial guide for future real-world implementations in SEN settings. Overall, the manuscript reads very well, there are only minor issues to be addressed. Please consider our points as suggestions, mainly raised to improve readability for researchers not very familiar with implementation science.

Minor issues

Introduction

Overall: the introduction is quite long, but I really like it because it explains in detail some key aspects of implementation science which are relevant for the paper. However, I feel that some content of the methods section could add the introduction (CFIR, ERIC, IOF, COM-B, SEM). I encourage the authors to re-think the introduction, and incorporate the descriptive parts of the methods into the introduction.

Further, please think about adding some kind of a “flow diagram” or figure/overview of the different frameworks and models you utilize, and how they are linked in your approach. Especially readers, that are not that familiar with implementation science will benefit from that.

Methods

Overall: if you explain the frameworks, models,… utilized, I would suggest to always start with the (specific) aim of each method, and then explain how it was utilized.

L233: how many people were approached overall, and also with respect to the three groups? Please add the response rates. Did you have the chance to randomize?

Line 238: The wording “The aim was to recruit…” here leads to the question if that aim was achieved or if it was a desired criterion that you did not achieve.

Line 248: It was not clear to me whether every participant was required to have a consultation or whether this was voluntary.

Line 256-259: As you had 2 workshops with a different focus, you could elaborate on the content of each workshop in the text, not just in the figure.

Line 320-324: You could add who participated in which workshop (distribution groups/ gender).

Strengths and Limitations

Line 489-494: I would suggest providing more background to the specific SEN setting in Northern Ireland here or in the introduction. How is PA typically included in SEN settings? Which SEN settings are you referring to? Schools that only carry to special educational needs? inclusion schools? Providing this information helps readers identify to what extent these findings could be applicable to their context.

I think it is also important to mention earlier that your study only focuses on settings for intellectual and developmental disabilities (as opposed to physical disabilities or mental health challenged children). The first time this is mentioned is in line 245.

Figure 1: do you really need a figure here? Please consider adding more information to the figure, e.g. some key aspects/tasks etc. that were addressed within each workshop. At the moment, the figure is not working if standing alone. Further, I suggest to order the legend according to the appearance of the frameworks, not in an alphabetical order.

Figure 2: I am afraid that figure 2 is too busy, it does not read well if printed or online.

Reviewer #3: Title: Embedding Behavioural and Implementation Science in a Whole School Approach to

Promote Physical Activity Among Children and Young People with Disabilities

Overall Statement: Thank you for the opportunity to review this manuscript. This manuscript aimed to develop an implementation research logic model to guide the adoption of a whole school approach to physical activity within special educational needs settings. The community-based participatory approach, involving parents, teachers, and other community stakeholders throughout the formulation, strengthens the IRLM’s relevance and potential for real-world impact. I believe the findings can have a profound impact on school-based physical activity promotion, particularly among children and young people living with disabilities. I have provided detailed, section-by-section comments for the authors that I believe will strengthen the paper.

Abstract

• The number of barriers and facilitators identified from workshop discussions does not seem to add up to the number of implementation determinants

• Similar to a previous comment, the authors state that 8 strategies were developed but only list 3. If you are using these as an example they should be placed in parentheses (e.g., staff training, building collaborative networks, engaging families).

Background

• There appears to be an extra space between lines 72-73 between sentences 2 & 3 that should be removed.

• The introduction to Whole of School Approaches is good, but Lines 71-72 stick out from the rest of the paragraph. These lines would be better suited for inclusion in paragraph 3 (lines 81-95), or as the transition sentence at the end of paragraph 2.

• The subheading for Paragraph 3 (lines 81-95) does not align with the text. Most of Paragraph 3 provides information that overlaps and/or compliments paragraph 2 (Overview of what a Whole of School Approach is). There needs to be much more than 1 sentence that discusses what’s been done in schools as it pertains to physical activity and special education/needs

• Lines 106-108, particularly “considering the unique contextual needs,” should be elaborated on; otherwise, the reader is left assuming what these are

• Line 113: it is difficult to identify which factors contributed to successful adoption and implementation, as a WSA is not only adopted but implemented too. The authors state implementing earlier in the sentence but it should be included in both places

• Delete comma in line 128

• Line 135-136 seems to be missing words or has a misplaced comma.

• Lines 138-141 should be simplified further. The aim currently is too wordy and is not communicated effectively enough.

• Making Objective 1 more concise would improve clarity; the remaining objectives are well-written

Methods

• Line 249 about purposive sampling should be introduced 1-2 paragraphs earlier in the recruitment section as it conflicts with the previous line about those who were already invited to participate

• The authors state the workshops were guided by the IRLM. Was there anything done to help parents/guardians understand implementation science and theories/frameworks?

• It is unclear who did the initial coding – was this AB & EB or someone else? Did the coders code independently or together? Did the coders ever meet to check the consistency in their codes? How were differences in codes resolved?

• Line 293-294: were all determinants successfully mapped to CFIR or was this the categories of determinants that were grouped together? Also, the final sentence reads like Results rather than Methods.

• Line 307: there are many things being referred to as determinants (contextual and implementation factors, barriers and facilitators). I recommend greater consistency throughout the paper

Results

• The barrier and facilitator counts add up to 32 determinants, not 31. Is there one missing?

• Organizing the example outcomes by level (individual, school, etc.) within the far-right column would help the reader understand the level at which these are operating

• Line 351-353: Move this to Methods

• There is a lack of information provided about the implementation outcomes. It was difficult to see the text in the figures provided, but since these were deductively coded, information on how frequently these outcomes were mentioned should be available. This is something that could be reported to strengthen the Results

• This section flips back and forth between active and passive voice

• How many people attended each workshop?

Discussion

• Line 385 is too vague (“several unique contextual challenges”). Since it was not provided in the first paragraph of the discuss, it is recommended that the authors provide an overview/recap of what those unique challenges. This will help the reader understand what they are compared later in the paragraph when an overview of existing literature is presented.

• Recent work by Craig et al. (https://doi.org/10.1186/s12966-025-01815-7 & https://doi.org/10.1186/s12889-024-17744-2) has also found some similar results when studying implementation of a whole of school approach, albeit lacking a focus on the special needs context.

• The extra space between lines 388 and 389 should be removed.

• Lines 397-391: This is good, but could be better connected with the paragraph above. Another option is to include how schools address these factors. The current structure of the discussion with subheadings actually makes the narrative read less connected. In its current form, this section of text is just a rewrite of the results.

• Lines 413-417: One sentence should not be a standalone paragraph. This should follow a similar structure as the paragraph immediately before and after.

• Lines 425-427: Citations are needed

• Limitations: The small sample is a major limitation. In particular, there were very few parents represented (n=2) and a limited amount of those who work in schools every day (n=4), and who will be in charge of implementing interventions, that participated.

• Limitations: There was no mention of who attended each workshop and whether they were balanced. Power dynamics between EANI reps and parents could have been present and may have influenced who spoke, how often, and what the responses were.

• Limitations: How many schools were represented? There were 4 teachers, 1 classroom assistant, and 2 from school leadership teams, but were all of these from different schools?

Other

• The text within the figures was extremely blurry in multiple places, including all of Fig 2, and could not be read

**********

what does this mean?). If published, this will include your full peer review and any attached files.

If you choose “no”, your identity will remain anonymous but your review may still be made public.

**Do you want your identity to be public for this peer review?**  For information about this choice, including consent withdrawal, please see our Privacy Policy

Reviewer #1: No

Reviewer #2: No

Reviewer #3: No

**********

[NOTE: If reviewer comments were submitted as an attachment file, they will be attached to this email and accessible via the submission site. Please log into your account, locate the manuscript record, and check for the action link "View Attachments". If this link does not appear, there are no attachment files.]

To ensure your figures meet our technical requirements, please review our figure guidelines: https://journals.plos.org/plosone/s/figures

You may also use PLOS’s free figure tool, NAAS, to help you prepare publication quality figures: https://journals.plos.org/plosone/s/figures#loc-tools-for-figure-preparation.

NAAS will assess whether your figures meet our technical requirements by comparing each figure against our figure specifications.

Attachment Submitted filename: PLOS One 3.22.26.docx

# Author comment: Author response to Decision Letter 1

**DOI:** 10.1371/journal.pone.0362562.r002

14 Oct 2026

See detailed response to reviewer feedback in uploaded document.

Attachment Submitted filename: Response to Reviewers PLOSOne.docx

# Editor report: Decision Letter 1

**DOI:** 10.1371/journal.pone.0362562.r003

**Authors:** Branko Aleksic

28 Oct 2026

Embedding Behavioural and Implementation Science in a Whole School Approach to Promote Physical Activity Among Children and Young People with Disabilities

PONE-D-26-02300R1

Dear Dr. Byrd,

We’re pleased to inform you that your manuscript has been judged scientifically suitable for publication and will be formally accepted for publication once it meets all outstanding technical requirements.

Within one week, you’ll receive an e-mail detailing the required amendments. When these have been addressed, you’ll receive a formal acceptance letter and your manuscript will be scheduled for publication.

An invoice will be generated when your article is formally accepted. Please note, if your institution has a publishing partnership with PLOS and your article meets the relevant criteria, all or part of your publication costs will be covered. Please make sure your user information is up-to-date by logging into Editorial Manager at Editorial Manager® and clicking the ‘Update My Information' link at the top of the page. For questions related to billing, please contact billing support.

If your institution or institutions have a press office, please notify them about your upcoming paper to help maximize its impact. If they’ll be preparing press materials, please inform our press team as soon as possible -- no later than 48 hours after receiving the formal acceptance. Your manuscript will remain under strict press embargo until 2 pm Eastern Time on the date of publication. For more information, please contact onepress@plos.org.

Kind regards,

Branko Aleksic, MD, PhD

Academic Editor

PLOS One

Additional Editor Comments (optional):

Reviewers' comments:

# Editor report: Acceptance letter

**DOI:** 10.1371/journal.pone.0362562.r004

**Authors:** Branko Aleksic

PONE-D-26-02300R1

PLOS One

Dear Dr. Byrd,

I'm pleased to inform you that your manuscript has been deemed suitable for publication in PLOS One. Congratulations! Your manuscript is now being handed over to our production team.

At this stage, our production department will prepare your paper for publication. This includes ensuring the following:

* All references, tables, and figures are properly cited

* All relevant supporting information is included in the manuscript submission,

* There are no issues that prevent the paper from being properly typeset

You will receive further instructions from the production team, including instructions on how to review your proof when it is ready. Please keep in mind that we are working through a large volume of accepted articles, so please give us a few days to review your paper and let you know the next and final steps.

Lastly, if your institution or institutions have a press office, please let them know about your upcoming paper now to help maximize its impact. If they'll be preparing press materials, please inform our press team within the next 48 hours. Your manuscript will remain under strict press embargo until 2 pm Eastern Time on the date of publication. For more information, please contact onepress@plos.org.

You will receive an invoice from PLOS for your publication fee after your manuscript has reached the completed accept phase. If you receive an email requesting payment before acceptance or for any other service, this may be a phishing scheme. Learn how to identify phishing emails and protect your accounts at https://explore.plos.org/phishing.

If we can help with anything else, please email us at customercare@plos.org.

Thank you for submitting your work to PLOS One and supporting open access.

Kind regards,

PLOS One Editorial Office Staff

on behalf of

Dr Branko Aleksic

Academic Editor

PLOS One
